# Randomized controlled trial of a smartphone-based cognitive behavioral therapy for chronic tinnitus

**DOI:** 10.1371/journal.pdig.0000337

**Published:** 2023-09-07

**Authors:** Uso Walter, Stefan Pennig, Tanja Kottmann, Lothar Bleckmann, Kristina Röschmann-Doose, Winfried Schlee

**Affiliations:** 1 ENT Practice Walter & Zander, Duisburg and mynoise GmbH, Duisburg, Germany; 2 context, Essen-Kettwig, Germany; 3 CRO Dr. med. Kottmann GmbH & Co KG, Hamm, Germany; 4 ENT Practice Dr. med. Bleckmann, Kleve, Germany; 5 G. Pohl-Boskamp GmbH & Co. KG, Hohenlockstedt, Germany; 6 Eastern Switzerland University of Applied Sciences, St. Gallen, Switzerland; 7 Clinic and Polyclinic for Psychiatry and Psychotherapy, University of Regensburg, Regensburg, Germany; McGill University, CANADA

## Abstract

Tinnitus, the phantom perception of sounds, generates distress and anxiety in those affected. Cognitive behavioral treatment approaches reproducibly help patients in managing chronic tinnitus. This study systematically evaluated the usefulness of a tinnitus app (with areas of attention and relaxation, mindfulness, acceptance, self-efficacy), which is prescribed for a total of nine months. One hundred eighty-seven participants with chronic tinnitus were equally randomized to an intervention arm that used a smartphone-based intervention -marketed as Kalmeda Tinnitus app-. and a control arm with delayed onset of treatment by 3 months. The first 3 months of a 9-months prescribed intervention have been analyzed as primary outcome. The Tinnitus Questionnaire (TQ) was used as primary endpoint to determine the reduction of tinnitus distress. Following intervention, there was a statistically significant and clinically relevant reduction of the TQ sum score in the intervention group compared to the control group (p<0.001, Cohen’s d effect size = 1.1). The secondary parameters, Patient Health Questionnaire-9 (PHQ9) and Perceived-Stress-Questionnaire (PSQ20) scores improved significantly in the intervention group whereas the Self Efficacy-Optimism-Pessimism short form (SWOP-K9) scores remained unchanged in both groups. Patients reported no treatment-related side effects. Taken together, use of this Tinnitus app lead to a significant decrease in tinnitus distress and a clinically relevant effect in the patients´ self-reported everyday management.

## Author summary

Hearing sounds and noises that are not there can be very distressing to the individual affected with tinnitus. Novel treatment approaches include psychotherapeutic applications, which are well received by patients, including their online delivery. A Smartphone app for treating tinnitus has been developed based on the latest know-how in behavioral therapy and we have tested its safety and effectiveness in 187 patients with tinnitus (at least 3 months´ duration). Tinnitus related distress and associated burden was significantly decreased in the intervention group compared to the control group expressed by significant reductions of TQ as well as PHQ9 and PSQ20 scores. In contrast, SWOP-K9 scores did not change in any of the groups.

## Introduction

Tinnitus is defined as the perception of an acoustic signal in the absence of an external auditory stimulus and has a complex origin. In European countries the prevalence of tinnitus was found to be 14.7%, with wide variation between countries [1].

The extent to which tinnitus causes distress is related to the patient´s selective attention and amplification of negative thoughts and is shaped by comorbidities and certain personality traits. Somatic, psychological, and social factors interact and are associated with the development of chronic tinnitus [2].

In a cross-sectional study tinnitus patients were found to show resilience in the face of experiencing tinnitus. Psychosocial factors shaped their self-rated health, and were found to be modifiable via enhancement of self-management, competence, and social activity. The training content may be delivered by the health practitioner with or without the aid of ehealth solutions [3].

Advances in understanding neural signaling have been made through electrophysiological measurements of patients suffering from tinnitus, and more recently, functional magnetic resonance imaging. Together they corroborate the view that neural connectivities are altered in such a way that they are postulated as the root cause of developing tinnitus in those who are susceptible and for whom tinnitus can be debilitating. These measurable changes affect the central auditory system, the limbic system, the functional default mode network as well as attention and control systems. Together, these studies represent an important breakthrough in the understanding why specific drug treatment is not yet available and why the application of cognitive behavioural approaches in the treatment of „bothersome tinnitus”is justified [4].

A clear demand for innovative technological advances to satisfy patients´ needs has been identified, especially in areas where treatment provision is perceived as not readily available or too sparsely distributed across regions [5]. Physician and patient led evaluation of new technological applications are needed in order to objectify the usefulness of these developments [5]. In patients with tinnitus, factors amenable to primary care intervention using an ehealth approach, as estimated from a population-based study, were suggested to be: aiding with sleep disturbance, increasing self-management, regaining control, and enhancing social activity. The hypothesis was that the population diagnosed with tinnitus would increase their resilience by using carefully designed behavioral modules and that the incidence of bothersome tinnitus would be diminished [3]. Self-help formats of cognitive behavioral therapy (CBT) appear to have a place in the self-management of patients with tinnitus whose access to in-person treatment is limited or who prefer to engage in their own time with their illness and symptoms via an individually accessible platform [6].

In the symptomatic treatment of tinnitus, the rationale for CBT has been to bring into consciousness the negativity of automatic responses to tinnitus and to change these into a positive connotation; more recently, acceptance-commitment therapy—a concept evolved from CBT—has been applied to patients with tinnitus where patients learn to accept and work through their negative reactive patterns in a process based therapy that incorporates long-term goals [7]. At present, CBT can be regarded as the intervention with most evidence for efficacy in reducing tinnitus distress [8,9]. In the treatment of tinnitus, long term benefits (at last 6 months) of acceptance and commitment therapy have been reported; a key determinant was tinnitus acceptance [10]. Internet-delivered acceptance and commitment therapy was shown to be effective in a systematic review of randomized controlled trials conducted for the treatment of anxiety, including tinnitus [11]. Sound treatment in the management of tinnitus has been shown in a network meta-analysis to be the singular most effective treatment that had a significant impact on perceived severity and quality of life. Combining sound treatment with tinnitus retraining, educational information or drug therapy further increased the success rate, as demonstrated across the 20 eligible studies [12].

Kalmeda Tinnitus app was conceived to represent five areas of different psychoeducational focus: redirecting attention and relaxation—these two are intensively reinforced over 3 months -, mindfulness, acceptance, and self-efficacy. Over a total period of 7–9 months, patients learn to recognize detrimental patterns of thought and behavior and to turn these to their benefit: they learn to identify personal needs and values to derive a set of goals, tapping own resources to achieve these goals or asking others for help and accepting help when offered. Importantly, patients learn to meet their thoughts and feelings, even the unpleasant/ negative / disquieting ones, with an attitude of mindfulness and acceptance and thereby develop resilience in the management of their condition. In addition, there are calming nature and background sounds, exercises to relax and meditate as well as tinnitus related information.

The rationale of this study was to investigate whether a cognitive behavioral approach—strongly advised (in its various forms) in German good practice guidelines [13]—can be safely and effectively delivered using the Kalmeda Tinnitus app. Therefore, a randomized interventional clinical study was conducted and the main objective of this study was to investigate the clinical efficacy of the smartphone-based cognitive behavioral in patients with chronic tinnitus that either received the app (intervention group) or waited for this period before receiving the app (control group). Both groups used the Tinnitus app for 9 months in total. The hypothesis was that a positive health care effect would be achieved by the study population when using the Tinnitus app for at least 3 months.

## Results

### Patient demographics and disposition

**Fig 1:**
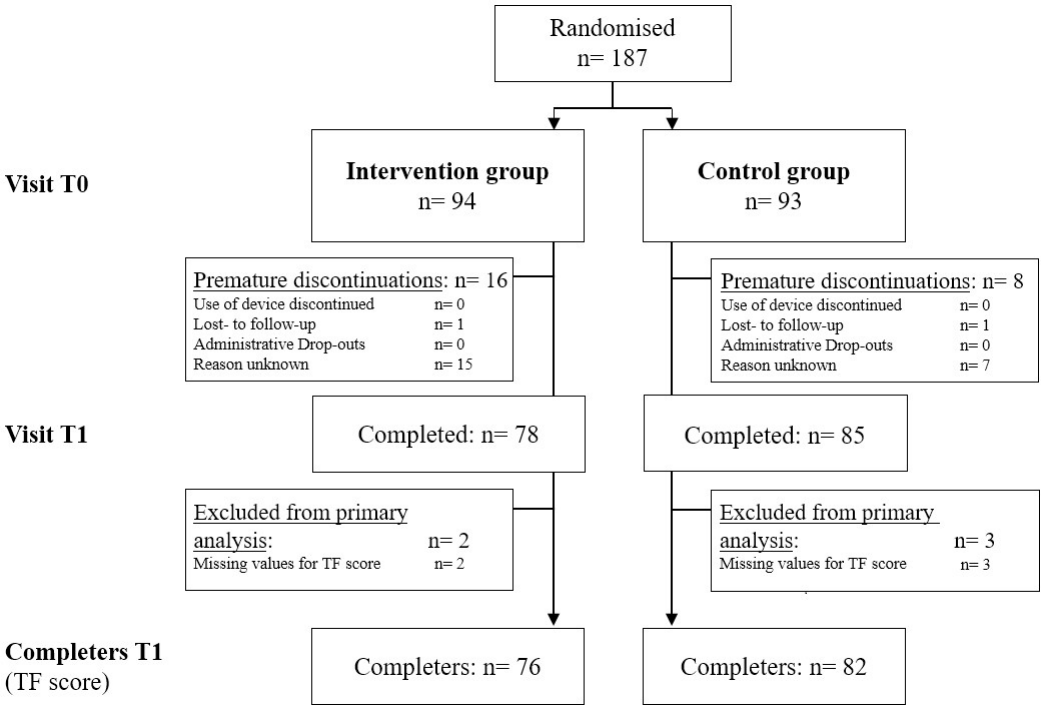
Disposition of patients; n = number.

187 patients were block-randomized to an intervention group which began using the app straight away, and a control group which provided the untreated control assessment timepoint after 3 months before start using the app (Fig 1). This population was evaluated for primary analysis.

The average age was 48.2 ± 12.5 years (females: 47.4 ± 12.1, males: 49.4 ± 12.7), with 97 (51.9%) male and 90 (48.1%) female participants, and duration of tinnitus was on average 6.57 ± 6.93 years for the entire cohort. There were no significant differences between the groups regarding baseline demographic data and baseline parameters assessed using the study questionnaires (S1 Table).

Premature discontinuations after 3 months´ use of the Tinnitus app added up to 16/94 (17.1%) patients in the intervention group and 8/93 (8.6%) patients in the control group, respectively (Fig 1). In total, 163 patients completed the study without premature discontinuation and filled out the Tinnitus Questionnaire (TQ) at least once (Fig 1). Completers had documented values for the primary parameter (TQ sum score) at T0 (baseline) and T1 (after 3 months of therapy); the intervention group accounted for 76 completers, the control group for 82 completers, 158 patients in total.

### Usage of Tinnitus app significantly improves subjective tinnitus burden

The mean durations of the study intervals were not significantly different between the groups: the mean duration (± SD) of the 3 months treatment period was 93 ± 12 days for the control group and 98 ± 12 days for the intervention group.

The TQ comprises 52 items scored by the patients, 6 sub-scales and 2 sum scales. Evaluation of TQ is based on internationally agreed standard instructions [14], and 40 of the 52 items are assigned to the 6 subscales. The calculation of the TQ sum score is based on the addition of all sub-scale scores, indicating a tinnitus related psychological distress sum score. Missing values at T1 (18/94 in the intervention group; 11/93 in the control group) were replaced according to baseline-based multiple imputation.

T0 values of the Patient’s TQ sum score (mean ± SD) were 39.7 ± 15.1 and 38.3 ± 15.1 in intervention and control group, respectively, and did not differ significantly. At T0 the proportion of patients with decompensated tinnitus (grade 3 or 4, corresponding to a TQ sum score of at least 47 points [13]) was comparable between control and intervention group (control: 28/93 patients = 30.1%; intervention: 29/94 patients = 30.9%). 3 months of treatment with the Kalmeda Tinnitus app induced a significant reduction in the proportion of patients with a decompensated tinnitus (13/94 patients = 13.8%; missing data imputed with baseline observation carried forward, BOCF), in contrast no reduction in severity of tinnitus was observed within the control group (30/93 patients = 32.3%; missing data imputed with BOCF, Chi^2^: 4.3, p<0.05).

**Fig 2:**
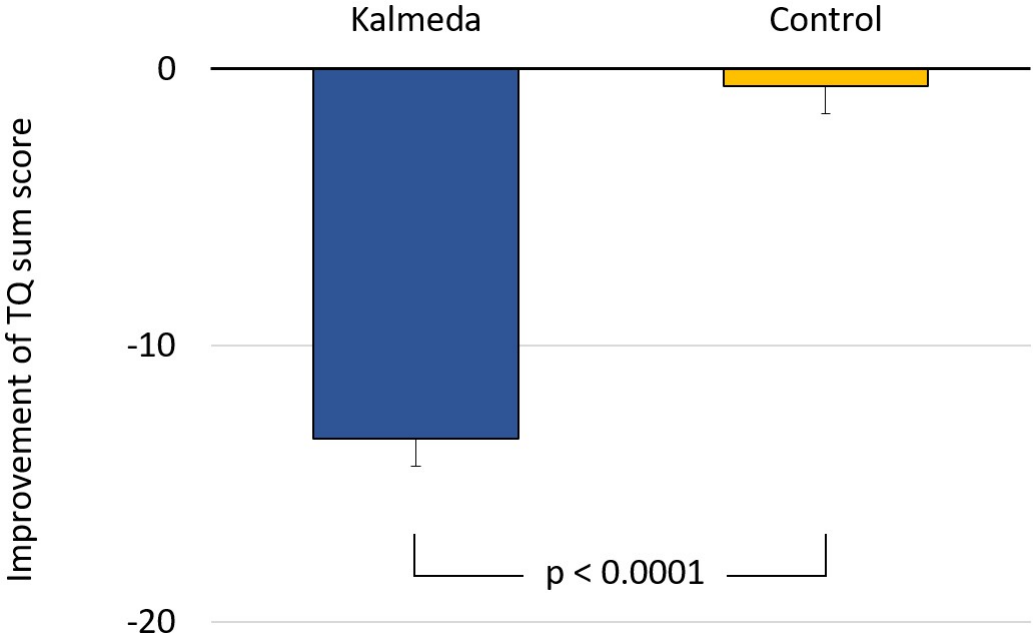
Change of the primary endpoint Tinnitus Questionnaire (TQ) sum score based on the intention to treat (ITT) population of patients (imputation: multiple imputations).

In the analysis of the primary endpoint, the model-adjusted treatment difference (with multiple imputation) between the intervention group and the control group was -12.74 ± 1.38 score points (ANCOVA; LS-Mean ± SE) in favor of the intervention group, which was statistically significant with p<0.0001 (Table 1, Fig 2).

**Table 1:** ANCOVA of the primary endpoint: Tinnitus Questionnaire (TQ) sum score (baseline based multiple imputation).

Parameter	Estimate	Standard error	p-value	95% confidence interval	
				Lower limit	Upper limit
Intercept	5.47	2.17	0.0120	1.21	9.73
Intervention	-13.36	1.00	<0.0001	-15.34	-11.39
Control	-0.63	0.98	0.5166	-2.55	1.28
Baseline	-0.16	0.05	0.0029	-0.26	-0.05
Intervention—control	**-12.74**	**1.38**	**<0.0001**	**–15.43**	**-10.03**

The additional variance-analytical evaluation showed for the differences T1-T0 within the intervention group an improvement of the TQ sum score by -13.36 ± 1.00 score points (ANCOVA; LS-Mean ± SE; p< 0.0001), while the TQ sum score in the control group showed no statistically significant differences in the same period with -0.63 ± 0.98 score points (ANCOVA; LS-Mean ± SE; p = 0.517; Table 1).

This demonstrates that there was a significant reduction in tinnitus burden (psychological distress or somatic reactions caused or aggravated by tinnitus) in the intervention group compared to the control group at T1 (p<0.0001).

Additional sensitivity analyses in ITT with BOCF and reference baseline-based imputations as well as in the data set of completers did not show significant differences (S2 and S3 Tables).

Moreover, the significant improvement in the intervention group was also observed for the intervention in all subscores when compared to the control group (emotional (F: 42.95, p< .0001) and cognitive burden (F: 43.47, p< .0001) -groupable as mental burden- (F: 50.58, p< .0001), loudness of tinnitus (F: 30.20, p< .0001), acoustic problems (F: 17.41, p< .0001), sleep deprivation (F: 25.48, p< .0001) and somatic complaints (F: 3.98, p< .05);). Using the non-adjusted mean difference of the primary endpoint between intervention and control group, calculation of Cohen´s effect size d resulted in 1.10 (95%CI [0.79, 1.41]).

The comparison of number of patients either with improvement of the TQ score between T0 and T1 larger than the minimal clinically important difference (MCID) of -6.65 or worsening of symptoms was performed for completers in intervention and control group. In the intervention group, in 56 of 76 (73.7%) patients TQ improved by more than -6.65 but only in 18 of 82 completers (22%) in the control group. 6 patients (7.9%) in the intervention group reported worsening of symptoms at T1. In 40 patients (48.8%) of the control group the TQ score increased during the study period. The number of patients with clinical important improvements (Chi^2^: 42.4 p = .0001) or worsening of symptoms (Chi^2^: 31.95 p = .0001) were significantly beneficial in the intervention group when compared to the control group.

### Usage of Tinnitus app significantly lowers perception of stress, depression and increases self-efficacy

Whether the use of Kalmeda Tinnitus App had a beneficial effect on areas relevant to patients with tinnitus outside the actual tinnitus burden was assessed as secondary parameters. There was a significant decrease in severity of depression by means of the PHQ-9 summative score for patients of the intervention group (decrease of 1.3 ± 2.4 score points) while there was no improvement for the control group (increase of 0.3 ± 2.4 score points; ANCOVA, LS-mean, p < .001; imputation BOCF, S4 Table). Cohen´s effect size d was 0.66 (95% CI [0.36–0.95]), which corresponds to a moderate effect.

Similarly, subjective perception of stress with Perceived-Stress-Questionnaire (PSQ-20) summative score was significantly reduced in the intervention group at T1 (-4.2 ± 11.9 score points), while there was no significant change for the control group over the same time (-0.5 +/- 9.5 score points, S5 Table). Cohen´s effect size d was 0.35 (95% confidence interval [0.06, 0.64]), which corresponds to a rather small effect.

In contrast, self-efficacy, as assessed by the Self Efficacy-Optimism-Pessimism short form (SWOP-K9) questionnaire was not a discriminatory parameter after 3 months of treatment for intervention and control groups. Initial values in intervention and control group were 2.8 and did not change in either group (p>0.05, S6 Table).

### Adverse events

There were no adverse events reported during the first 3 months of study duration.

## Discussion

This randomized controlled trial pursued the hypothesis that use of the Tinnitus app would alleviate tinnitus and its associated disease burden through stepwise engagement of the participant with modular content specifically developed for this condition.

Evidence based digital health applications are in the process of transforming the landscape of clinical care, aided, in Germany, by the Digital Health Care Act passed in late 2019. They are thought to be instrumental in overcoming perceived barriers for those patients who find access to specialist care difficult and/or who benefit from regular illness specific guidance when experiencing tinnitus [15]. Patients with tinnitus are a group for whom this gap in provision may be effectively and safely filled by a smartphone app that incorporates scientific evidence in the delivery of its modules.

CBT has been shown to be effective at decreasing the impact of chronic tinnitus on the perceived reduction in quality of life by patients (in comparison to a waiting group control). For this outcome, the standardized effect size derived from ten studies was -0.56 (95% confidence interval -0.83, -0.30) [16]. Based on this evidence, CBT is strongly recommended in the national guidelines on the treatment of chronic tinnitus as this approach showed no risk and has the potential to achieve habituation in the patients [13].

The main objective of this study was to investigate the clinical efficacy of the smartphone-based cognitive behavioral treatment element delivered by the patients´ use of Kalmeda Tinnitus app. Patients that participated in the study had a moderate tinnitus score (median TQ score of 39), and after 3 months´ use (primary endpoint), there was a significant improvement for the intervention group: In comparison to the control group, this group showed a significant reduction in tinnitus associated distress as measured using the TQ. Within the intervention group the observed improvement of TQ Score by use of the Tinnitus app exceeded the MCID of -6.65 reported by a study linking TQ Scores and clinical global expression [17]. The calculated Cohen’s d of 1.10 (95% CI: 0.79; 1.41) corresponds to a strong effect of the intervention, which is in line with current literature. In a meta-analysis values obtained from TQ were linked with the patient-reported outcome in subjective clinical global impression (CGI) to determine differences, that are clinically relevant. The study revealed that changes of TQ score and CGI ratings were well correlated and changes of– 5 and + 1 were identified as MCID for improvement and worsening, respectively [17]. Furthermore, in a study that investigated the effect of an internet-based cognitive behavioral therapy (iCBT) between-group effect size of Cohen`s D was evaluated to be 0.76 for the Tinnitus Functional Index (TFI) [18].

The sensitivity analyses (completer and replacing missing data in 2 additional ways) proved consistency with the results of the primary analysis and excluded a major influence of baseline imbalances. It might well be that the moderate severity of tinnitus distress prevailing in this study made the patients more amenable to psychotherapeutic management approaches [19]. This assumption is supported by a secondary analysis of iCBT studies in tinnitus, which revealed that the education level and the baseline severity of tinnitus distress are significant predictor variables for the therapy`s effectiveness, confirming the impact of the disease’s severity and further demographic factors [20]. The authors conclude that heterogenous populations should be preferred in order to avoid any bias. However, within the study reported here, the education level of patients was not documented, but as recruitment of patients was performed online without geographical restrictions, the population investigated appeared to be representative for the indication.

The Tinnitus app investigated addresses aggravating consequences of tinnitus, such as developing depression and managing stress. There was a significant improvement in scores that measure depression and stress, indicating that the use of the Tinnitus app alleviated these expressed features of chronic tinnitus.

Available evidence shows a positive impact of CBT, including acceptance and commitment therapy on the management of tinnitus, which improved the perceived quality of life, not on the perception of tinnitus. However, a final decision on long term efficacy (as of 6 months) was still pending [16]. A retrospective evaluation of patient`s feedback after iCBT clearly confirms the importance of acceptance, change in own behavior, and patient`s commitment for treatment success [21].

Our results show that already after 3 months of CBT via Tinnitus app, depression and stress perception were significantly improved, pointing towards an increase in quality of life, though there was no difference at this timepoint in the self-assessed areas self-efficacy and optimism. It is possible that the 3 months intervention phase was too short to have a significant effect on this parameter, which could be detected in the 9 months data or that no causal relationship with tinnitus burden exists [22].

While it appears that by using Kalmeda Tinnitus app, the patient partakes in his or her treatment with a certain autonomy, the somewhat greater retention of the control group may be related to the additional contact the control group had with the study team. Their shorter study duration supports this notion, being reflective of anticipatory enthusiasm resulting in obviously higher adherence to the envisaged time frame. Study drop-out rate—though expected [15]—was small. A previous meta-analysis suggested the presence of a significant placebo effect on tinnitus distress when on the waiting list for cognitive behavioral treatment [23]. This was not observed in the present study, as baseline and 3 months´ evaluation of TQ were not significantly different.

Although conducted as randomized RCT this study displays some limitations. In contrast to other studies [24] the use of the Kalmeda Tinnitus app was not completely guided by an audiologist, but patients had the opportunity to contact a principal investigator in the case of any questions. Synergism of CBT via app and parallel guidance by trained audiologists might increase the overall impact of treatment, but was not subject of the underlying clinical study. iCBT, when compared to individualized face-to-face CBT proved to be equally effective in reducing tinnitus distress [25]. In another study that compared the outcome of iCBT with face-to-face group CBT the authors evaluated that iCBT might be the preferred treatment of choice for tinnitus patients that are open towards new forms of therapy and who are willing to work autonomously [26]. This is in line with an evaluation of factors that facilitate the application of new interventions in the treatment of tinnitus [18]. The authors found that the availability of iCBT and the motivation of patients are key factors for a positive outcome and impact of treatment in the reduction of tinnitus distress. The results obtained with the Kalmeda Tinnitus app, where patients worked autonomously, and regained control over their burden by learning to modify perception and behaviour and to increase acceptance, confirm these findings. A follow-up interview was not part of the study reported here, but would be a useful tool to further evaluate patient`s needs in order to optimize the app. Due to the nature of treatment no placebo control or blinding were possible. Bias in evaluation however was excluded by blinded data management. Besides the initial use of the code for downloading the app no monitoring of actual use was performed due to interference with national data protection regulation. For future studies statements on the actual use of the app provided by the patients e.g. via eDiary entries should be implemented. Concomitant therapy was not documented throughout the study; however, parallel use of CBT was excluded. In order to comply with national regulations concerning the duration of the study, the primary endpoint was defined to be evaluated after 3 months of therapy, rather than considering the individual tempo of the patients while absolving the levels, which could have been achieved with a level-based endpoint. Taken together our study shows that the Kalmeda Tinnitus app is effective in patients suffering compensated and decompensated tinnitus. The reduction of tinnitus distress was found to be significant and clinically relevant, when compared to current literature. To the best of our knowledge this app is the first iCBT for the treatment of tinnitus that was granted reimbursement in Germany. The longevity of the app-based treatment effect will be assessed in an evaluation, which covers the whole 9 months treatment phase.

## Materials and methods

### Patients and legal framework

The clinical investigation plan was approved by the Ethics Committee Nordrhein, Germany on May 20, 2020 (case number 2020026). Recruitment started in August 2020; the last patient was included in March 2021. The study was conducted in two referral practices in North Rhine Westphalia, Germany, under the auspices of CRO Dr.med. Kottmann GmbH & Co. KG, following SOPs on on-site and remote monitoring, data management, and data safety, in the context of the Digital Care Act and using pseudonymized patient data.

Patients browsing the landing website of the Kalmeda app were invited to ascertain their eligibility to take part in the study, if interested. Criteria for inclusion were 18 years of age and older, chronic subjective tinnitus of more than 3 months´ duration [13]; for exclusion, diagnosed acute or chronic mental illness. The participants were then sent via mail the informed consent form, and the consenting process was finalized during a videochat with a physician for the confirmation of diagnosis and control of in- and exclusion criteria. The patients posted the signed informed consent form and were randomized to one of the treatment arms. Questionnaires were sent in time for the 3 and 9 months´ timepoints. There were recruitment drives over social media such as facebook, twitter and youtube. Self-help groups of the German Tinnitus League and members of the state-wide otorhinolaryngological network were informed by email, and the study was announced in the sponsor’s newsletter.

### Study design

The study was registered on 09.10.2020 with the German Clinical Trials Register (DRKS: access ID is DRKS00022973). The concept of the study followed the ISO 14155 norm (Clinical investigation of medical devices for human subjects—Good clinical practice) and recommendations contained in „Evidence Standards Framework for Digital Health Technologies”for Tier C digital health products (NICE, 2021). By using a web based Electronic Data Capture (EDC) System (secuTrial, interactiveSystems, Berlin, Germany) patients were consecutively in the order of their appearance randomized in blocks of six to either an intervention arm that began using the app straight away at T0 (intervention group) or a control arm that waited for 3 months until the primary endpoint was reached by the patients of the invention arm (T1), before themselves start using the app. Both groups continued to use the app for a total of 9 months, but analyses reported here are restricted to the first 3 months (intervention vs. untreated control). Data covering the full 9 months intervention period are going to be published after analysis is completed. The validated questionnaires that were used in this study were Tinnitus questionnaire (TQ) to quantify tinnitus distress [27], Patient health questionnaire-9 (PHQ-9) to capture depression [28], Perceived-stress-questionnaire (PSQ20) to assess subjective stress [29] and Self efficacy-optimism-pessimism short form (SWOP-K9) for self-efficacy and optimism [30]. Data was entered in the web based electronic data capture system.

### Kalmeda Tinnitus app and associated regulations

Developed by Mynoise GmbH, Duisburg, Germany, Kalmeda app was designed to follow principles of CBT (self-management following the Zürcher Ressourcen Modell ZRM (motivational psychology) and acceptance and commitment therapy (Westin et al., 2011; Ost, 2014)). It is CE marked and was placed on the market as a medical device in 2019 (DIMDI registration number: DE/CA20/mynoise_01/18). The software version that was used in the study (iOS at least 9.0, Android at least 6.0) was 1.5.1.

The app consists of five levels with nine steps each. Levels 1 and 2 of the behavioral therapy (areas redirecting attention, relaxation) are typically completed in 3 months. Subsequent levels 3–5 deal with the areas of mindfulness, acceptance, self-efficacy and are typically completed in not less than 7 months. Exercises are complemented by soothing nature and background sounds, guided meditation, and tinnitus related information. A therapist was available for questions that did not include medical advice.

### Statistical analyses

The statistical analyses were performed using the statistical software SAS 9.4 (TS1M6) for Microsoft Windows. Sample size calculations were based on an estimated effect size of 0.5, a power of 80% and a level of significance of 0.05, as conducted by two-sided t-test. This resulted in 64 patients per group, so 128 in total. A drop-out rate of 15% was assumed and 150 patients were originally planned for recruitment. Because there was a possibility that the waiting group design could lead to a reduction in observed effect size (Hasford and Koch, 2017) and a higher dropout rate over the nine months of total duration of therapy per patient, which may limit generalizability of the results, a larger recruitment volume was striven for. A non-stratified block randomization strategy was followed to randomly assign blocks of six patients in consecutive order to the two study arms.

The primary endpoint was defined as the treatment difference between intervention and control groups calculated from the mean differences using model-adjusted multiple imputations to account for missing values of the TQ sum score between baseline (T0) and 3 months (T1).

Furthermore, treatment related changes in the following parameters were assessed in all app-users after 3 months: depression (PHQ-9), stress perception (PSQ20) and self-efficacy (SWOP). Missing values were substituted using Baseline Observation Carried Forward (BOCF). For the primary endpoint, the null hypothesis was tested by covariance analysis (ANCOVA), with changes in tinnitus questionnaire total score being the dependent variable, intervention group as independent variable, and baseline value being the covariate. For the analyses of secondary endpoints, differences between intervention and control group at T0 and T1 were analyzed using paired and unpaired t-test.

Missing values were replaced with values following the multiple imputation principle. Reasons for missing values were premature discontinuations, missing questionnaires (n = 24), or missing answers to questionnaire items (n = 5). Sensitivity analyses were performed for the primary endpoint to test for robustness of the primary analysis [31]. This was done via 3 methods to test the null hypothesis of the primary endpoint: i. imputation using the baseline observation carried forward method (BOCF; n = 187), ii. analysis of complete data sets (76 patients of the intervention group and for 82 patients of the control group, the “completers in t1”, i.e., patients for whom there is TQ sum score at baseline (T0) and at 3 months (T1), and iii. baseline-based multiple imputation (where 25 datasets were generated by regression analysis using baseline-based pattern imputation). To detect the group effect between intervention and control group in the change of TQ sum score comparing T0 and T1, an ANCOVA was used, with the change of TQ sum score as dependent factor and the baseline values as covariate.

## Supporting information

S1 Table **A.** Characteristics of enrolled participants. **B.** Enrolled participants´ baseline questionnaire scores. The individual areas assessed in TQ and PSQ20 are weighted equally for the calculation of their sum scores.
(DOCX)

S2 Table Tinnitus Questionnaire sum score (BOCF). (DOCX)

S3 Table ANCOVA sensitivity analysis of primary endpoint, Tinnitus Questionnaire sum score (BOCF). (DOCX)

S4 Table PHQ-9 summative score (BOCF). (DOCX)

S5 Table PSQ-20 summative score (BOCF). (DOCX)

S6 Table SWOP-K9 summative score (BOCF). (DOCX)

S7 Table Subject data listings questionnaires. (XLSX)
