# Supplementary material for: Randomized controlled trial of a smartphone-based cognitive behavioral therapy for chronic tinnitus
Source: PLOS Digit Health. 2023 Sep 7;2(9):e0000337. doi: 10.1371/journal.pdig.0000337 (PMC10484427; doi:10.1371/journal.pdig.0000337)
Supplement: S1 Table — A. Characteristics of enrolled participants. B. Enrolled participants´ baseline questionnaire scores. The individual areas assessed in TQ and PSQ20 are weighted equally for the calculation of their sum scores. (DOCX) [file pdig.0000337.s001.docx]

**S1 Table** Characteristics of ITT participants and baseline questionnaire scores

1. Characteristics of ITT participants.

|  | **intervention group** | **control group** | **Statistical Test** |
| --- | --- | --- | --- |
| Participants: n | 94 | 93 |  |
| Males: n (%) | 45 (47.9%) | 52 (55.9%) | Chi²-test: p=0.2711 |
| Females: n (%) | 49 (52.1%) | 41 (44.1%) |  |
| Age: mean ± SD  (range) | 48.1 ± 12.8  (22- 72) | 48.4 ± 12.2  (21- 74) | t-test: p=0.8555 |
| Tinnitus Duration in years: mean ± SD (range) | 6.21 ± 6.62  (0.33-28) | 6.94 ± 7.25  (0.25-31) | U-test: p=0.7914 |

1. Enrolled participants´ baseline questionnaire scores.

|  | **intervention group** | **control group** | **Statistical Test** |
| --- | --- | --- | --- |
| TQ at Baseline  [mean ± SD]  emotional burden  cognitive burden  penetrance  acoustic problems  sleeping problems  somatic complaints  Sum score | 11.04 ± 5.18  7.80 ± 3.52  10.59 ± 3.15  4.44 ± 3.34  3.68 ± 2.28  2.11 ±1.78  39.65 ±15.08 | 10.73 ± 5.14  7.44 ± 4.06  10.38 ± 3.27  4.52 ± 3.47  2.90 ± 2.35  2.33 ±1.89  38.30 ± 15.10 | t-test: n.s. |
| PHQ-9 at Baseline  [mean ± SD] | 8.31 ± 3.89 | 7.53 ± 4.21 | t-test: n.s. |
| PSQ-20 at Baseline  [mean ± SD]  Worries  Tension  Joy  Challenge  Sum score | 35.89 ± 20.09  57.38 ± 21.67  50.78 ± 19.16  45.18 ±23.65  46.91 ±17.56 | 33.91 ± 20.18  55.27 ± 23.66  52.19 ± 22.21  43.58 ± 24.97  45.14 ± 18.89 | t-test: n.s. |
| SWOP-K9 at Baseline  [mean ± SD]  Self-efficacy  Optimism  Pessimism | 2.76 ± 0.45  2.76 ± 0.77  2.05 ± 0.69 | 2.75 ± 0.52  2.78 ±0.72  2.01 ± 0.80 | t-test: n.s. |
