# Supplementary material for: Randomized controlled trial of a smartphone-based cognitive behavioral therapy for chronic tinnitus
Source: PLOS Digit Health. 2023 Sep 7;2(9):e0000337. doi: 10.1371/journal.pdig.0000337 (PMC10484427; doi:10.1371/journal.pdig.0000337)
Supplement: S2 Table — (DOCX) [file pdig.0000337.s002.docx]

**S2 Table** Tinnitus Questionnaire sum score (BOCF)

|  | **ITT Intervention group** | | | **ITT Control group** | | |
| --- | --- | --- | --- | --- | --- | --- |
|  | **baseline** | **at three months** | **Δ** | **baseline** | **at three months** | **Δ** |
| n | 94 | 94 | 94 | 93 | 93 | 93 |
| Median, Range [min; max] | 39 [12 ; 73] | 25 [2 ; 73] | -11 [-42 ; 8] | 36 [9 ; 76] | 37 [2 ;76] | 0 [-25 ; 18] |
| Mean ± SD  [95% confidence interval] | 39.65 ± 15.08  [36.56, 42.74]) | 28.85 ± 17.27  [25.31, 32.39] | -10.8 ± 10.12  [-12.87,-8.73]^a^ | 38.30 ± 15.10  [35.19 , 41.41] | 37.63 ± 16.30  [34.28 , 40.99] | -0.67 ± 8.19  [-2.35, 1.02]^n.s.^ |
