# Supplementary material for: Randomized controlled trial of a smartphone-based cognitive behavioral therapy for chronic tinnitus
Source: PLOS Digit Health. 2023 Sep 7;2(9):e0000337. doi: 10.1371/journal.pdig.0000337 (PMC10484427; doi:10.1371/journal.pdig.0000337)
Supplement: S3 Table — (DOCX) [file pdig.0000337.s003.docx]

**S3 Table** ANCOVA sensitivity analysis of primary endpoint, Tinnitus Questionnaire sum score (BOCF)

| **Parameter** | **Estimate** | **Standard error** | **p-value** | **95% confidence interval** | |
| --- | --- | --- | --- | --- | --- |
|  |  |  |  | **Lower limit** | **Upper limit** |
| Intercept | 1.90 | 1.96 | 0.3324 | -1.96 | 5.77 |
| Intervention | -10.75 | 0.95 | <0.0001 | -12.62 | -8.88 |
| Control | -0.71 | 0.95 | 0.4555 | -2.59 | 1.17 |
| Baseline | -0.07 | 0.04 | 0.1350 | -0.16 | 0.02 |
| Δ Intervention - Control | **-10.04** | **1.34** | **<0.0001** | **–12.69** | **-7.39** |

Δ, value at 3 months (T1) – value at baseline (T0)
