# Supplementary material for: Randomized controlled trial of a smartphone-based cognitive behavioral therapy for chronic tinnitus
Source: PLOS Digit Health. 2023 Sep 7;2(9):e0000337. doi: 10.1371/journal.pdig.0000337 (PMC10484427; doi:10.1371/journal.pdig.0000337)
Supplement: S4 Table — (DOCX) [file pdig.0000337.s004.docx]

**S4 Table:** PHQ-9 summative score (BOCF)

|  | **ITT Intervention group** | | | **ITT Control group** | | |
| --- | --- | --- | --- | --- | --- | --- |
|  | **baseline** | **at three months** | **Δ** | **baseline** | **at three months** | **Δ** |
| N | 94 | 94 | 94 | 93 | 93 | 93 |
| Range [min; max] | [2; 19] | [0;19] | [ -; 3] | [0; 21] | [0; 21] | [-1; 8] |
| Average ± SD | 8.3 ± 3.9 | 7.0 ± 3.8 | -1.3 ± 2.4 | 7.5 ± 4.2 | 7.8 ± 4.2 | 0.3 ± 2.4 |
| paired t-test | t(93) = 5.15, p<.0001 | |  | t(92) = -1-15, p=.2546 | |  |
| t-test **Δ** | t = -4.55, p < .0001 | | | | | |
