# Supplementary material for: Randomized controlled trial of a smartphone-based cognitive behavioral therapy for chronic tinnitus
Source: PLOS Digit Health. 2023 Sep 7;2(9):e0000337. doi: 10.1371/journal.pdig.0000337 (PMC10484427; doi:10.1371/journal.pdig.0000337)
Supplement: S5 Table — (DOCX) [file pdig.0000337.s005.docx]

**S5 Table:** PSQ-20 summative score (BOCF)

|  | **ITT Intervention group** | | | **ITT Control group** | | | |
| --- | --- | --- | --- | --- | --- | --- | --- |
|  | **baseline** | **at three months** | **Δ** | **baseline** | **at three months** | **Δ** | |
| N | 94 | 94 | 94 | 93 | 93 | 93 | |
| Range [min; max] | [11.7; 95.0] | [1.7; 95.0] | [-60.0; 23.3] | [11.7; 83.3] | [1.7; 83.3] | [-28.3; 25.0] | |
| Average ± SD | 46.9 ± 17.6 | 42.7 ± 18.1 | -4.2 ± 11.9 | 45.1 ± 18.9 | 44.6 ±20.8 | -0.5 +/- 9.5 | |
| Paired t-test | t(93) = 3.45, p=.0008 | |  | t(92) = 0.51, p=.6117 | |  | |
| t-test **Δ** | t = -2.37, p = .0188 | | | | | |  |
