# Supplementary material for: Randomized controlled trial of a smartphone-based cognitive behavioral therapy for chronic tinnitus
Source: PLOS Digit Health. 2023 Sep 7;2(9):e0000337. doi: 10.1371/journal.pdig.0000337 (PMC10484427; doi:10.1371/journal.pdig.0000337)
Supplement: S6 Table — (DOCX) [file pdig.0000337.s006.docx]

**S6 Table:** SWOP-K9 summative score (BOCF)

|  | **ITT Intervention group** | | | **ITT Control group** | | |
| --- | --- | --- | --- | --- | --- | --- |
|  | **T0** | **T1** | **Δ** | **T0** | **T1** | **Δ** |
| **SWOP efficacy** |  |  |  |  |  |  |
| N | 94 | 94 | 94 | 93 | 93 | 93 |
| Range [min; max] | [1.8; 4.0] | [1.2; 4.0] | [-0.80; 0.80] | [1.4; 3.8] | [1.4; 3.8] | [-0.60; 1.40] |
| Average ± SD | 2.76 ± 0.45 | 2.82 ± 0.51 | 0.03 ± 0.28 | 2.75 ± 0.52 | 2.79 ±0.53 | 0.06 ± 0.32 |
| **ANCOVA** | F: 0.34, p = 0.56 | | | | | |
| **SWOP optimism** |  |  |  |  |  |  |
| N | 94 | 94 | 94 | 93 | 93 | 93 |
| Range [min; max] | [1.0; 4.0] | [1.0; 4.0] | [-1.50; 2.00] | [1.0; 4.0] | [1.0; 4.0] | [-1.50; 1.00] |
| Average ± SD | 2.76 ± 0.77 | 2.81 ± 0.74 | 0.08 ± 0.50 | 2.78 ± 0.72 | 2.73 ±0.70 | -0.02 ± 0.46 |
| **ANCOVA** | F: 2.15, p = 0.14 | | | | | |
| **SWOP pessimism** |  |  |  |  |  |  |
| N | 94 | 94 | 94 | 93 | 93 | 93 |
| Range [min; max] | [1.0; 3.5] | [1.0; 4.0] | [-1.00; 1.00] | [1.0; 3.5] | [1.0; 3.5] | [-2.00; 1.00] |
| Average ± SD | 2.05 ± 0.69 | 1.99 ± 0.72 | -0.01 ± 0.48 | 2.01 ± 0.80 | 2.03 ± 0.77 | -0.01 ± 0.56 |
| **ANCOVA** | F: 0.26, p = 0.87 | | | | | |
